# The *Leptospermum scoparium* (Mānuka)-Specific Nectar and Honey Compound 3,6,7-Trimethyllumazine (Lepteridine^TM^) That Inhibits Matrix Metalloproteinase 9 (MMP-9) Activity

**DOI:** 10.3390/foods12224072

**Published:** 2023-11-09

**Authors:** Bin Lin, Smitha Nair, Daniel M. J. Fellner, Noha Ahmed Nasef, Harjinder Singh, Leonardo Negron, David C. Goldstone, Margaret A. Brimble, Juliet A. Gerrard, Laura Domigan, Jackie C. Evans, Jonathan M. Stephens, Troy L. Merry, Kerry M. Loomes

**Affiliations:** 1 School of Biological Sciences and Institute for Innovation in Biotechnology, The University of Auckland, Auckland 1142, New Zealand; blin040@aucklanduni.ac.nz (B.L.); smitha.nair@auckland.ac.nz (S.N.); d.goldstone@auckland.ac.nz (D.C.G.); m.brimble@auckland.ac.nz (M.A.B.); j.gerrard@auckland.ac.nz (J.A.G.); 2 School of Chemical Sciences, The University of Auckland, Auckland 1142, New Zealand; dfel694@aucklanduni.ac.nz; 3 Riddet Institute, Massey University, Palmerston North 4410, New Zealand; n.nasef@massey.ac.nz (N.A.N.); h.singh@massey.ac.nz (H.S.); 4 Callaghan Innovation, Gracefield Innovation Quarter, 69 Gracefield Road, Lower Hutt 5010, New Zealand; leonardo.negron@ginanbio.com; 5 Maurice Wilkins Centre for Molecular Biodiscovery, The University of Auckland, Auckland 1142, New Zealand; troy.merry@comvita.com; 6 Department of Chemical and Materials Engineering, The University of Auckland, Auckland 1142, New Zealand; l.domigan@auckland.ac.nz; 7 Comvita NZ Limited, 23 Wilson Road South, Bay of Plenty, Paengaroa 3189, New Zealand; jackie.evans@comvita.com (J.C.E.); jmcstephens@outlook.com (J.M.S.); 8 Discipline of Nutrition, School of Medical Sciences, The University of Auckland, Auckland 1142, New Zealand

**Keywords:** *Leptospermum scoparium*, Mānuka honey, inflammation, gastric ulcers, Lepteridine, matrix metalloproteinase 9, MMP-9

## Abstract

3,6,7-trimethyllumazine (Lepteridine™) is a newly discovered natural pteridine derivative unique to Mānuka (*Leptospermum scoparium*) nectar and honey, with no previously reported biological activity. Pteridine derivative-based medicines, such as methotrexate, are used to treat auto-immune and inflammatory diseases, and Mānuka honey reportedly possesses anti-inflammatory properties and is used topically as a wound dressing. MMP-9 is a potential candidate protein target as it is upregulated in recalcitrant wounds and intestinal inflammation. Using gelatin zymography, 40 μg/mL Lepteridine^TM^ inhibited the gelatinase activities of both pro- (22%, *p* < 0.0001) and activated (59%, *p* < 0.01) MMP-9 forms. By comparison, Lepteridine^TM^ exerted modest (~10%) inhibition against a chromogenic peptide substrate and no effect against a fluorogenic peptide substrate. These findings suggest that Lepteridine^TM^ may not interact within the catalytic domain of MMP-9 and exerts a negligible effect on the active site hydrolysis of small soluble peptide substrates. Instead, the findings implicate fibronectin II domain interactions by Lepteridine^TM^ which impair gelatinase activity, possibly through perturbed tethering of MMP-9 to the gelatin matrix. Molecular modelling analyses were equivocal over interactions at the S1′ pocket versus the fibronectin II domain, while molecular dynamic calculations indicated rapid exchange kinetics. No significant degradation of synthetic or natural Lepteridine^TM^ in Mānuka honey occurred during simulated gastrointestinal digestion. MMP-9 regulates skin and gastrointestinal inflammatory responses and extracellular matrix remodelling. These results potentially implicate Lepteridine^TM^ bioactivity in Mānuka honey’s reported beneficial effects on wound healing via topical application and anti-inflammatory actions in gastrointestinal disorder models via oral consumption.

## 1. Introduction

Oral consumption and topical application of honey is used in the treatment of a wide range of health ailments including gastrointestinal disorders and healing of wounds and ulcers [1]. The effectiveness in treating these conditions may relate to honey’s reported antibacterial, antiproliferative, anti-inflammatory, and antioxidant properties [2]. Mānuka honey, derived from the nectar of *Leptospermum scoparium* (Mānuka) trees native to Aotearoa New Zealand, is renowned for its potent non-peroxide antibacterial activity and wound healing properties [3] and is a high-value food product used by consumers for a range of conditions including the treatment of cough and cold symptoms and digestive health [4,5]. A key component of Mānuka honey’s non-peroxide antibacterial activity is methylglyoxal (MGO), which is derived from the spontaneous conversion of dihydroxyacetone (DHA) present within the *Leptospermum scoparium* nectar [6]. This unique non-peroxide antibacterial activity together with high osmolarity and acidic matrix contribute to Mānuka honey’s reported wound healing properties.

However, Mānuka honey is also replete in polyphenols such as phenolic acids and flavonoids and other bioactive compounds [7,8,9,10]. The therapeutic contribution of these small molecule components is likely under-appreciated given their potential anti-inflammatory and radical scavenging actions [11]. Indeed, MGO activity alone is unlikely to explain the bioactivity and anti-inflammatory effects of orally consumed Mānuka honey in rat disease models of inflammatory bowel disease (IBD) and gastric ulcer [12,13,14]. This is because it is apparent that MGO is rapidly degraded during gastrointestinal digestion, and consumption of dietary MGO exerts no influence on MGO concentrations assessed in vivo or in the urine [15,16].

3,6,7-trimethyllumazine (trademarked Lepteridine^TM^) is a pteridine derivative that is unique to Mānuka honey [7,17]. While the structural assignment of Lepteridine^TM^ has been confirmed by total synthesis [7], its biological function and potential molecular targets are yet to be elucidated. Pteridines support a host of biological processes ranging from being enzymatic co-factors to forming animal pigments. Because of these diverse biological activities, there is substantial interest in the therapeutic potential of pteridine derivatives [18]. Many pteridine derivatives attenuate bacterial lipopolysaccharide-induced inflammatory cytokine responses in vitro [19,20], and the pteridine derivative analogue, 4AZA1378, attenuates symptoms of trinitrobenzenesulphonate (TNBS)-induced IBD in mice [21]. Notably, the pteridine derivative medicine, methotrexate, is commonly prescribed for the treatment of IBD and other auto-immune and inflammatory diseases such as rheumatoid arthritis and psoriasis [21].

Matrix metalloproteinases (MMPs) belong to a family of over 20 structurally related zinc-dependent proteases [22]. They possess a signal peptide, pro-peptide, active site, zinc-binding domain, and a C-terminal hemopexin domain (except MMP-7, 26 and 23) linked to the catalytic domain through a hinge region. MMP-2 and -9 contain a fibronectin type II domain in addition to these domains and play a crucial role in every stage of wound healing by regulating the balance between tissue synthesis and destruction [23]. However, excessive proteolytic activity, especially of MMP-9, results in non-healing chronic wounds and gastric ulcers via the degradation of a newly formed extracellular matrix and other required proteins [24]. This dysregulation of MMP-9 can lead to exaggerated and prolonged inflammation and has been postulated to underpin the intestinal inflammation that characterizes a host of inflammatory bowel diseases [25,26,27,28,29]. Thus, controlled activity of MMP-9 appears to be essential for systematic wound healing and intestinal epithelial health.

MMP-9, also known as gelatinase-B, readily digests denatured collagens and gelatins and is a potent basement membrane degrading enzyme, making it of particular interest for chronic wound healing therapy [24]. Indeed, genetic and pharmacological inhibition experiments implicate MMP-9 in the attenuation of intestinal muscularis inflammation and postoperative ileus [29]. MMP-9 also promotes inflammation by regulating soluble proteins including protease inhibitors [30] and chemokines [31], thereby implicating MMP-9 as a prototypical target in inflammatory diseases [32].

Given the pathophysiological importance of MMP-9 in inflammatory states, we hypothesised whether some of the anti-inflammatory effects of Mānuka honey observed in wound healing, rat IBD [13,14], and gastric ulcer models [12] could be mediated through this target. Since Lepteridine^TM^ is a stable pteridine derivative unique to Mānuka honey (*Leptospermum*) and found at concentrations up to >50 mg/kg [7,17,33], we embarked on enzymatic analyses with MMP-9 to determine the inhibitory potential, followed by molecular docking studies, to identify putative binding interactions with MMP-9 and other inflammatory protein targets.

## 2. Materials and Methods

### 2.1. Physicochemical Properties

The global minimum geometry and corresponding wavefunction of Lepteridine^TM^ were calculated using the quantum mechanical Density Functional Theory, for which the ωB97X-D functional was employed in conjunction with the fully augmented triple-zeta Def2TZVPPD basis set, in addition to a CPCM implicit water solvent model. These calculations were carried out in Spartan’20.

### 2.2. Molecular Docking

**Figure A1:**
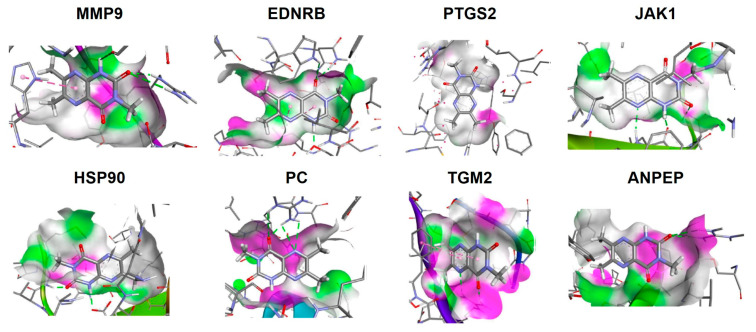
Highest-ranking docked pose for Lepteridine^TM^ within the catalytic active site of target protein candidates.

**Figure A2:**
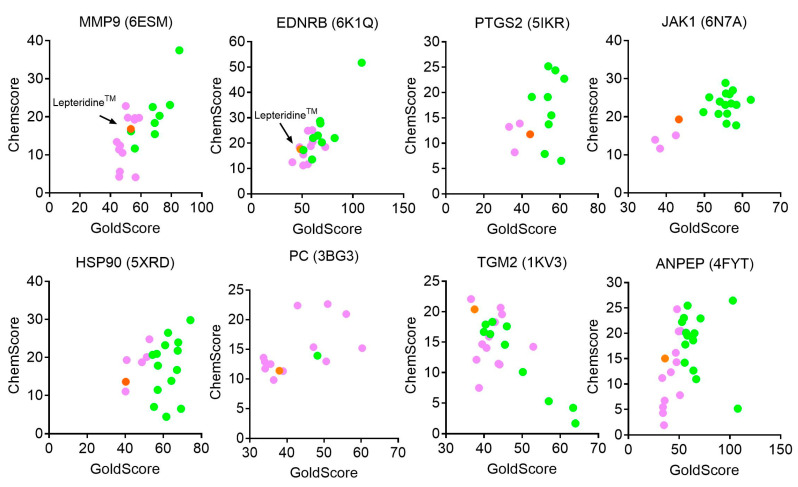
GoldScore and ChemScore distribution of reported poses for known ligands (green), non-ligands (purple), and Lepteridine^TM^ (orange).

**Figure A3:**
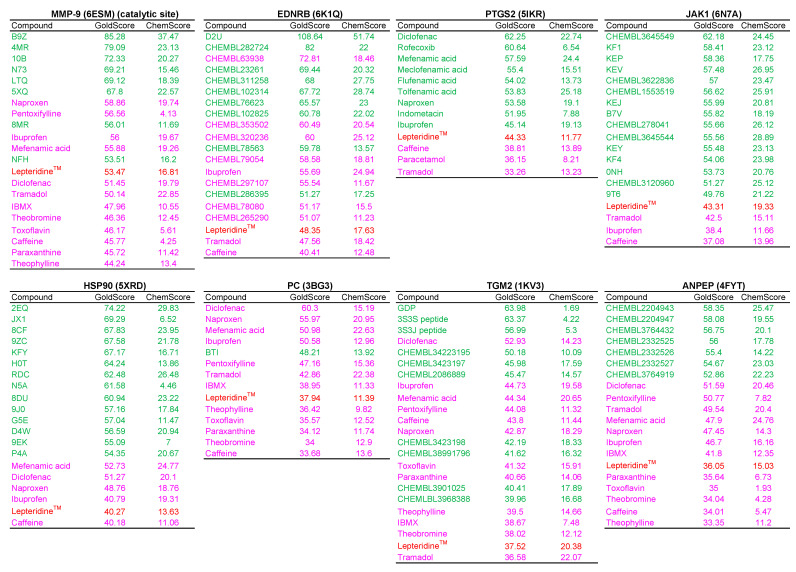
Scored poses for known ligands (green), Lepteridine^TM^, and non-ligands (pink) docking to protein targets.

Docking analyses between Lepteridine^TM^ and candidate inflammatory mediators (Table A1) were performed with GOLD v5.7.3, with a total of 10 GA runs per ligand and maximum search efficiency. Docked poses were scored with GoldScore and subsequently rescored with ChemScore. The top-ranking pose for Lepteridine^TM^ for each of the candidate proteins is shown in Figure A1. Co-crystallised ligands of known inhibitors to each of the candidate protein targets were used to validate pose prediction quality. Chemically or pharmacologically related ligands not known to possess affinity to these candidate proteins were also included as negative controls (Figure A2 and Figure A3). Crystallographically resolved structures were derived from the Protein Data Bank (PDB) (IDs in Table 1).

### 2.3. MMP-9 Fluorescent and Colorimetric Activity Assays

Lepteridine^TM^ and leptosperin were synthesised in house as described previously [7,34]. Compound purity was >95% by 1H NMR. Substrate cleavage-based fluorometric and colorimetric MMP-9 inhibitor screening assay kits were purchased from Abcam (Melbourne, Australia), which included recombinant MMP-9 enzyme, and the broad spectrum MMP-9 active site inhibitor, isobutyl-*N*-(4-methoxyphenylsulfonyl)glycyl hydroxamic acid (NNGH) [35]. Both fluorescent and colorimetric assays were performed as per the manufacturer’s instructions in a 96-well clear microplate included in the kit, with a final reaction volume of 100 µL. Prior to adding the supplied substrate, MMP-9 was incubated with either Lepteridine^TM^, leptosperin, or NNGH for 60 min at 37 °C. The reaction was then initiated through the addition of either the quenched fluorogenic or chromogenic substrate and allowed to run for 20 min or 120 min at 37 °C, respectively. Fluorescence was monitored at 328/420 nm (excitation/emission), while the colorimetric assay was monitored at 412 nm, corresponding to the production of 2-nitro-5-thiobenzoic acid.

### 2.4. Mass Spectrometry Activity Analyses

This experiment was designed as an orthogonal method to compare the relative abundance of the remaining quenched fluorogenic substrate after digestion by MMP-9 (Section 2.3). Four samples were prepared comprising (i) quenched fluorescent substrate only; (ii) quenched fluorescent substrate + MMP-9; (iii) quenched fluorescent substrate + MMP-9 + Lepteridine^TM^; and (iv) quenched fluorescent substrate + MMP-9 + NNGH. All samples were allowed to react for 30 min at 37 °C. Reactions were terminated by adding 1 µL of 50% acetic acid (*v*/*v*) and stored under dark conditions at −20 °C until analysis.

Substrate/metabolite were measured using a QSTAR XL hybrid Quadrupole-Time-of-Flight mass spectrometer (Applied Biosystems, Foster City, CA, USA). Samples were diluted 50-fold in 0.1% formic acid and a 10 μL injection was made into a 0.3 mm trap column at 30 μL/min for 3 min, followed by separation on a 0.3 × 100 mm Zorbax 300SB- C18 column (Agilent, Santa Clara, CA, USA). The HPLC gradient between Buffer A (0.1% formic acid in water) and Buffer B (0.1% formic acid in acetonitrile) was formed at 6 μL/min as follows: 10% B for the first 0.2 min, increasing to 75% B by 24 min, increasing to 97% B by 26 min, held at 97% until 29 min, back to 10% B at 30.5 min, and held there until 35 min. The LC effluent was directed into the ion spray source scanning from 330–1600 *m*/*z* for 1 s, followed by a 2-s product ion scan, fragmenting the protonated substrate ion of *m*/*z* 1093.5 with a collision energy of 80 V (*m*/*z* range 175–500). The mass spectrometer and HPLC system were under the control of the Analyst QS 2.0 software package (Applied Biosystems). Data analysis was carried out using PeakView v2.2 (Sciex).

### 2.5. Expression in E. coli, Denaturing Purification, and On-Column Refolding of Recombinant Human Matrix Metalloproteinase 9 (MMP-9) in Its Latent Form (His-Tagged proMMP-9cat)

cDNA encoding proMMP-9cat was cloned into a pET22 expression plasmid to yield a construct encoding an N-terminal His-tagged proMMP-9cat (pET22-His6-TEV-MMP-9-Pro-Cat) with ampicillin selection. The construct also encoded a TEV protease cleavage site between the Histidine tag and the pro-peptide domain of MMP-9. The pET22-His6-TEV-MMP-9-Pro-Cat plasmid was transformed into DH5α *E. coli* BL21 (DE3) cells according to standard procedures [36]. A single colony was picked from the overnight LB agar plate for induction and inoculated into 5 mL media (ZY) containing antibiotics and incubated overnight, with shaking at 37 °C. The overnight culture was inoculated (1:200) into fresh media (ZY5052) containing antibiotics. Harvested cells were lysed (20 mM Tris-HCl (pH 8)), 150 mM NaCl, 0.1% Triton, and 5 mM β-mercaptoethanol), incubated for 30 min on ice, and processed twice through a cell disrupter at 18 kPa, followed by centrifugation using a Sorval 62 S-34 rotor (Thermo Fisher Scientific, Waltham, MA, USA) at 8000× *g* for 30 min at 4 °C to separate soluble and insoluble fractions. The lysis pellet was used for the purification of inclusion bodies.

Following solubilization of inclusion bodies (6 M urea, 20 mM Tris-HCl (pH 8), 500 mM NaCl, 5 mM imidazole, and 0.5 mM tris(2-carboxyethyl)phosphine (TCEP) overnight, the denatured suspension was centrifuged to separate the insoluble debris. The supernatant containing denatured His-tagged proMMP-9cat was loaded using a peristaltic pump onto a 5 mL Immobilised Metal Affinity Chromatography (IMAC) column pre-equilibrated with solubilisation buffer (6 M urea, 1 mM TCEP, 0.5 M NaCl, 20 mM Tris-HCl (pH 7.4), and 20 mM imidazole). Following washing with detergent buffer (0.1 M NaCl, 20 mM Tris-HCl, 1 mM TCEP, and 0.1% Triton X-100), the IMAC column was washed first with cyclodextrin buffer (0.1 M NaCl, 20 mM Tris-HCl, 5 mM cyclodextrin, 5 mM CaCl_2_, 1 µM ZnCl_2_, 10 mM reduced glutathione, and 1 mM oxidised glutathione), followed by a column wash buffer (0.5 M NaCl, 20 mM Tris-HCl, 5 mM CaCl_2_, and 1 µM ZnCl_2_). His-tagged proMMP-9cat was eluted (0.1 M NaCl, 20 mM Tris-HCl, and 500 mM imidazole) and fractions pooled together and concentrated using Amicon^®^ Ultra-15 3K centrifugal filter devices (EMD Millipore, Billerica, MA, USA).

The concentrated fraction containing refolded proMMP-9cat was filtered (0.2 µm) and applied to a size exclusion chromatography column equilibrated at 0.5 mL/min with 20 mM Tris-HCl (pH 8), 150 mM NaCl, and 0.1 mM TCEP). An ÄKTA explorer coupled with a UV monitor and ÄKTA fractionator (GE Healthcare Bio-Sciences, Pittsburgh, PA, USA) was used to inject samples, pump liquid through the column, and monitor and collect protein factions (monitored at 280 nm). Fractions containing His-tagged proMMP-9cat were pooled and concentrated using a 3 KDa concentrator (~0.2 mg/mL), then flash frozen using liquid nitrogen and stored at −80 °C with 20% glycerol (final glycerol concentration).

### 2.6. Protein Quantitation

Purified His-tagged proMMP-9 was quantified immediately following purification with UV spectrophotometry using a NanoDrop^®^ ND-8000 UV-Vis Spectrophotometer (Thermo Fisher Scientific, Waltham, MA, USA). For biochemical assays, the total protein concentration was determined using the Pierce BCA assay [37], where the reduction in Cu^2+^ to Cu^+^ was monitored at 562 nm.

### 2.7. SDS/PAGE Electrophoresis and Western Blotting

Bacterial lysates or purified proteins were routinely analysed by SDS-PAGE to assess purity. Protein samples were added to a microcentrifuge tube containing 2× loading dye, incubated at 98 °C for 3 min to denature the samples, and then centrifuged prior to gel loading. Gels were run at 160 V, 275 mA for 40 min, stained using Coomassie blue solution for 30 min, and de-stained for 1 h. Gels were imaged using a Gel Doc™ XR+ Gel Documentation System (Bio-Rad, Hercules, CA, USA). For Western blot analyses, gels were transferred to a polyvinyl difluoride (PVDF) membrane using iBlot^®^ (Thermo Fisher Scientific, Waltham, MA, USA) and the membrane was then placed on an iBind™ Flex card (Thermo Fisher Scientific, Waltham, MA, USA) for antibody binding, according to manufacturer’s instruction.

### 2.8. Generation of Activated Human MMP-9cat from His-Tagged proMMP-9cat

After denatured IMAC purification and refolding, 250 µg refolded His-tagged proMMP-9cat was treated by trypsin to proteolytically separate the pro-domain from the catalytic, fibronectin, and zinc-binding domains [38]. The working trypsin concentration was 1:20, (trypsin–MMP ratio) with a 20 min incubation at 37 °C to obtain active MMP-9 now lacking the His-tagged pro-domain (MMP-9cat). Trypsin digestion following the 20 min incubation period was arrested immediately by the addition of ice-cold trypsin inhibitor (1:10, trypsin: inhibitor ratio, as per the manufacturer’s instructions). Cleavage of His-tagged proMMP-9cat to MMP-9cat was confirmed by SDS-PAGE under reducing conditions.

The activity of in-house activated MMP-9cat was performed using a commercial colorimetric assay as per the manufacturer’s instructions (BML-P125-005, Enzo Life Sciences, New York, NY, USA). Activated human MMP-9cat with or without the inhibitor, NNGH (20 µL), was equilibrated in assay buffer at 37 °C for 1 h. The reaction was initiated by the addition of 10 µL thiopeptide (100 µM final concentration) and monitored at 412 nm at 37 °C. An assay control with no MMP-9cat was maintained for each experiment. The activity of the MMP-9cat (mol substrate/min) was calculated using the extinction coefficient, 13,600 M^−1^cm^−1^, of the reaction product (2-nitro-5-thiobenzoic acid).

### 2.9. His-Tagged proMMP-9cat and MMP-9cat Gelatinase Activity Assay

Novex™ 10% Zymogram Plus (Gelatin) Protein Gels (15 wells) and Novex™ sharp pre-stained protein standard, Novex Tris-Glycine SDS sample buffer, Novex Tris-Glycine SDS running buffer, Novex zymogram renaturing buffer, and Novex zymogram developing buffer were all purchased from Thermo Fisher Scientific Inc. (Auckland, New Zealand). His-tagged proMMP-9cat was diluted to a final concentration of 5 μg/mL and gently mixed with loading buffer and water to achieve a total loading volume of 10 μL per well. Gel electrophoresis was performed using the XCell SurelockTM Mini-Cell system (Thermo Fisher Scientific, Auckland, New Zealand). The upper chamber was filled with 200 mL of 1X Tris-Glycine SDS running buffer and the lower chamber with 600 mL. The gel was run at a constant voltage of 125 V and 30 mA (starting current) for 105 min.

After electrophoresis, the gel was removed and incubated in 1X renaturing buffer for 30 min with gentle agitation. Following incubation, the gel was carefully cut into smaller pieces and further incubated separately in 1X developing buffer or Lepteridine^TM^-supplemented developing buffer for 30 min with gentle agitation. The gel was further incubated overnight for 12 h at 37 °C with a fresh developing buffer, with or without Lepteridine^TM^. NNGH was also added into the developing buffer at 2.6 μM as a positive inhibitor control. After incubation, the gelatin gel was rinsed with water three times (5 min each) with gentle agitation. The gel was stained by adding 20 mL of SimplyBlue Safestain and incubated for 2 h at room temperature with gentle agitation. It was destained by removing the SimplyBlue Safestain and rinsed with water for 2 h at room temperature. His-tagged ProMMP-9cat and MMP-9cat band activities were analysed using densitometry on ImageJ (Version 1.52a).

### 2.10. Lepteridine^TM^-Simulated Digestion

Mānuka honey samples with a range of different Lepteridine^TM^ concentrations were provided by Comvita New Zealand Ltd. The simulated gastrointestinal digestion was carried out using a static model. The simulated gastric fluid (SGF) and the simulated intestinal fluid (SIF) were prepared in accordance with a global consensus protocol [39]. SGF was maintained at pH 3 to mimic the fed-state of the stomach and a final mixture contained 2000 U/mL of pepsin. Two grams of raw Mānuka honey or synthetic Lepteridine^TM^ was incubated in 2 mL of SGF at 37 °C under 95 rpm shaking for a period of 2 h in triplicates. At 0, 30, 60, and 120 min of incubation, 0.1 mL of the mixture was withdrawn for Lepteridine^TM^ analysis. At 120 min, the remaining mixture was transferred to the SIF (pH 7) at a volume ratio of 1:1 (SIF:gastric digestion mixture), with a final concentration of 1 mg/mL of pancreatin and 10 mM of porcine bile extract. This mixture was incubated at 37 °C under 95 rpm shaking for up to 4 h in triplicate, and at 0, 60, 120, and 240 min, 0.1 mL of the mixture was withdrawn for Lepteridine^TM^ concentration analysis. The pancreatin activity in the withdrawn solution was quenched by adding 5 mmol/L Pefabloc^®^. SGF and SIF mixtures were diluted 5 times with 0.1% formic acid and then centrifuged at 14,000 rpm for 10 min.

Supernatant was recovered and analysed using a reverse-phase HPLC for Lepteridine^TM^ concentrations as described previously [7]. A Hypersil GOLD column (150 × 2.1 mm, 3 μM particle size) was used as the stationary phase (25 °C), and the mobile phase comprised 0.1% formic acid (phase A), and 80:20 acetonitrile: 0.1% formic acid (phase B). The injection volume was 3 μL, the flow rate was 0.200 mL, and a gradient elution as follows was used to separate Lepteridine^TM^ and others: initial 2 min (5% phase B), at 7 min (25% B), 14 min (50% B), 16 min (100% B), 19 min (5% B), and 20 min (5% B, held 10 min). The signal of Lepteridine^TM^ was detected at 320 nm.

### 2.11. Statistical Analysis

Statistical analyses were performed using GraphPad Prism software (Version 7.01) (GraphPad Software Inc., San Diego, CA, USA). One-way ANOVA was used to compare multiple groups’ means, followed by Tukey’s or Dunnett’s multiple comparison post-hoc test when the significance (*p*-value) was less than 0.05.

## 3. Results

### 3.1. Modelling of Lepteridine^TM^ as a Putative Ligand for MMP-9 and Other Inflammatory Mediators

**Figure 1:**
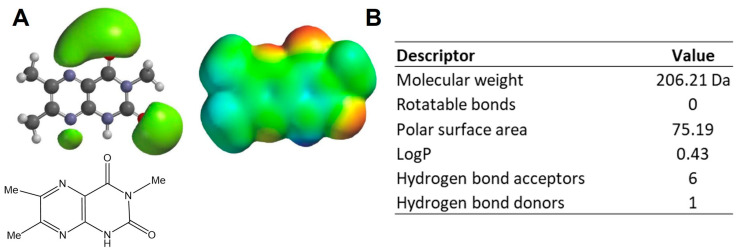
(**A**) Lepteridine^TM^ predicted structure and electrostatic potential. (**B**) Molecular descriptors as calculated using Spartan’22. LogP was calculated via the thermodynamic method using the CPCM Solvent model in conjunction with gas phase calculations of neutral and ionized species.

To investigate the potential for Lepteridine^TM^ to interact with MMP-9 and other inflammatory mediators, we examined its geometrics and electronics using a quantum mechanical Hartree–Fock method with the 3-21G basis set. Lepteridine^TM^ exhibited three distinct areas of negative electrostatic potential (Figure 1A), with a potential hydrogen bond pattern reminiscent of the adenine moiety of adenosine. Furthermore, the low molecular weight, compact, planar nature of the compound, and a logP value which is favourable for the addition of lipophilic functional groups (Figure 1A,B) indicates that Lepteridine^TM^ sits in the fragment-based drug range and would be expected to have multiple biological targets.

The catalytic domain of MMP-9 comprises the active site containing the catalytic Zn^2+^ ion, a conserved metzincin sequence motif, six binding pockets (S1, S2, S3 and S1′, S2′, S3′) that lie adjacent to the catalytic Zn^2+^ ion, and fibronectin domain clusters [40,41]. Molecular modelling was undertaken to assess the potential for Lepteridine^TM^ to dock with the S1′ pocket of MMP-9, which comprises a deep hydrophobic pocket that acts as a substrate recognition point. Given the fragment-like behaviour of Lepteridine^TM^ and potential for multiple targets, we expanded these analyses to other possible gastrointestinal inflammatory protein targets based on the literature (Table A1). GoldScore and ChemScore (Table 1) and the highest-ranking docked pose (Figure A1) for Lepteridine^TM^ with the active site of each candidate were calculated. Docking scores for Lepteridine^TM^ binding with each candidate were compared with docking scores of the candidate protein with other compounds of similar molecular weight and/or structures, with known activity or inactivity towards the candidate (Figure A2 and Figure A3).

In all cases, Lepteridine^TM^ was not predicted to strongly bind to any of the selected candidates’ active sites. Based on GoldScore and ChemScore, Lepteridine^TM^ showed similarities to known active ligands for MMP-9, JAK1, and TGM2, and of these, MMP-9 had the highest GoldScore (Table 1). The top-ranking pose for Lepteridine^TM^ to the MMP-9 protein structure 6ESM, which contains the primary catalytic S1′ pocket, shows H bond interactions between a carbonyl group and Arg249, and an H bond interaction between the H bond-donating nitrogen of Lepteridine^TM^ and a carbonyl group on the enzyme backbone (Figure A1). However, there is a clash between one of the pyrazine nitrogens and a backbone carbonyl oxygen, suggesting that Lepteridine^TM^ does not have appreciable affinity to MMP-9 at the catalytic domain.

**Figure 2:**
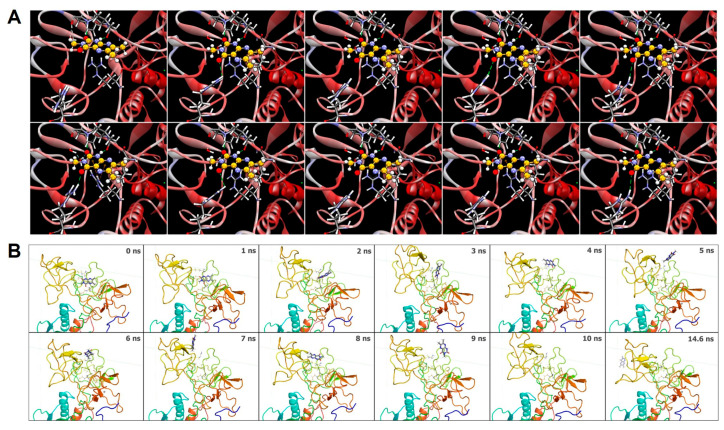
Molecular modelling of Lepteridine^TM^ at the fibronectin type II domain of MMP-9. (**A**) Top 10 returned binding poses of Lepteridine^TM^ using crystallographic structure, 1L6J, from the Protein Data Base. (**B**) Molecular dynamic snapshots of Lepteridine^TM^ bound to the fibronectin domain, resulting in dissociation after 10 ns.

MMP-9 also possesses exo sites within fibronectin II domains that can control its catalytic activity [42]. Molecular docking for the fibronectin domain using ChemScore, followed by rescoring with GoldScore, revealed multiple binding poses, the top 10 poses of which are shown in Figure 2A. For the optimal binding pose 1 (Chemscore: 18.94; GoldScore: 26.60), Lepteridine^TM^ is stabilized by the formation of two hydrogen bonds with the side chains of Arg332. The presence of oxygen, nitrogen (H-bond acceptor), and N-H group (H-bond donor) allows the formation of multiple hydrogen bonds between Lepteridine^TM^ and the fibronectin domain of MMP-9 with a distance of less than 2.5 Å in multiple different poses (1–10).

To gain an appreciation into the kinetic dynamics involved between Lepteridine^TM^ and the fibronectin domain, we ran molecular dynamics simulations (Figure 2B). Following optimization of CHARMM force field parameters, Lepteridine^TM^ was docked to the fibronectin domain of MMP-9. The simulation was conducted under conditions of water solvation with 0.15 M NaCl, with periodic boundary conditions where water wraps to the other side if it reaches the simulation cell boundary. Under these simulated conditions, Lepteridine^TM^ adopted different binding poses as soon as 2 nanoseconds and cycled through different binding pose modes before completely dissociating within 10 nanoseconds.

### 3.2. Fluorescence and Colorimetric Spectrometry Assessment of Lepteridine^TM^’s Ability to Inhibit MMP-9 Activity

**Figure 3:**
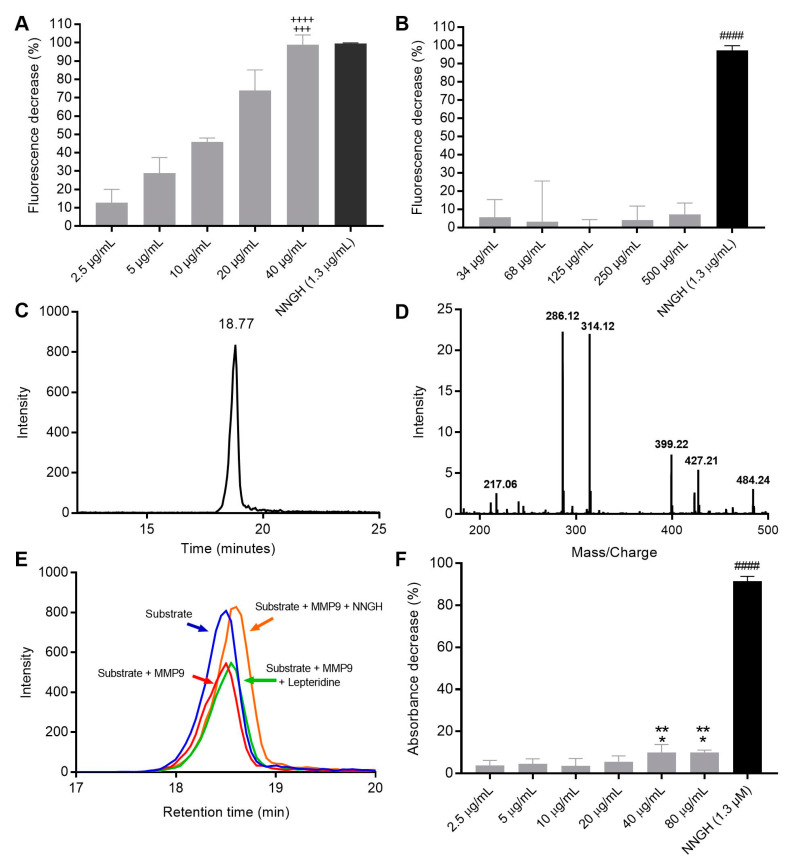
Effects of Lepteridine^TM^, leptosperin, and NNGH on MMP-9 activity as assessed using commercial fluorometric and colorimetric kits and mass spectrometry analyses. (**A**) Fluorescence-based assay of Lepteridine^TM^. ^++++^ *p* < 0.0001 versus 2.5, 5, and 10 μg/mL; ^+++^ *p* < 0.001 versus 20 μg/mL. (**B**) Fluorescence-based assay of leptosperin. ^####^ *p* < 0.0001 versus all concentrations. (**C**) Mass spectrum chromatographic peak(s) of intact MMP-9 quenched fluorogenic substrate and (**D**) its fragment ions. (**E**) Representative chromatographic peak areas extracted from the two most abundant fragments following incubation of recombinant MMP-9 and quenched fluorogenic substrate with and without Lepteridine^TM^ or NNGH. (**F**) Colorimetric substrate cleavage assay. ^####^ *p* < 0.0001 versus all concentrations: ** *p* < 0.01 versus 10 μg/mL; * *p* < 0.05 versus 2.5 and 5 μg/mL. Data show mean ± SEM (n = 3). Statistical significance was performed by one-way analysis of variance (ANOVA), followed by Tukey’s multiple comparison test.

Synthesised Lepteridine^TM^ was screened for MMP-9 inhibition using a commercial recombinant MMP-9 FRET-based assay. This assay relies on MMP-9 cleavage of a quenched fluorogenic peptide to liberate a non-quenched (7-methoxycoumarin-4-yl)-acetyl moiety. Following the 30 min assay incubation period, a dose-dependent decrease in fluorescent signal was observed for Lepteridine^TM^ at concentrations typically found in Mānuka honey (2.5–40 μg/mL), with a calculated IC_50_ of 11 μg/mL (Figure 3A). At 40 μg/mL, Lepteridine^TM^ decreased assay fluorescence by a similar (~99% decrease) amount as the commercial MMP-9 active site inhibitor, NNGH. By comparison, leptosperin, a unique methyl syringate glycoside derivative found in Mānuka honey, had no effect on MMP-9 activity at concentrations of 34–500 μg/mL, indicating some specificity for Lepteridine^TM^.

Since Lepteridine^TM^ itself has a fluorescent signal, and fluorescent compounds can interfere with fluorescent assays, we developed an orthogonal mass spectrometry assay and utilised an independent colorimetric assay to further interrogate Lepteridine^TM^ MMP-9 inhibition. Mass spectrometry analyses of the quenched fluorogenic peptide substrate used in the commercial fluorescent assay revealed a molecular weight of 1092.5 g/mol and retention time of 18.77 min (Figure 3C). The two most abundant fragment ions exhibited a *m*/*z* of 286.12 and 314.12 (Figure 3D). The total chromatographic peak area extracted from these two fragmented ions was used to assess the relative abundance of the intact quenched fluorogenic substrate, such that a lower peak area indicates higher MMP-9 activity. Interestingly, while the commercial MMP-9 inhibitor, NNGH, almost totally abolished MMP-9-induced cleavage of the intact fluorogenic substate, Lepteridine^TM^ had no effect on peak area over the 30 min incubation period (Figure 3E). This finding demonstrates conclusively that Lepteridine^TM^ does not have an inhibitory effect of MMP-9 on cleavage of the fluorogenic substrate, as indicated by the commercial fluorescence assay.

Due to inconsistencies in the results from fluorescence and mass spectrometry approaches, interactions between Lepteridine^TM^ and MMP-9 were further investigated using a commercial colorimetric assay. This assay relies on a chromogenic thiopeptide substrate that, when cleaved liberates a sulfhydryl group, reacts subsequently with Ellman’s reagent (5,5′-dithiobis(2-nitrobenzoic acid)) to generate 2-nitro-5-thiobenzoic acid, which can be detected at 412 nm. This chromogenic substrate provided by the commercial kit manufacturer was added into each well to initiate the reaction and the assay run for 120 min at 37 °C. These experiments indicated modest inhibition of MMP-9 activity at all Lepteridine^TM^ concentrations, amounting to a <10% decrease in activity, as compared to NNGH (Figure 3F). Nevertheless, inhibition by Lepteridine^TM^ was potentiated at both the 40 and 80 μg/mL concentrations compared to the 2.5 μg/mL and 10 μg/mL concentrations, consistent with a dose-dependent profile.

### 3.3. Purification and Characterisation of Recombinant proMMP-9cat

The weak MMP-9 inhibitory effect in the colorimetric assay suggested Lepteridine^TM^ might exert a non-competitive inhibitory effect on activity, which would be consistent with our molecular docking studies suggesting binding to the fibronectin type II domain. We therefore investigated the effects of Lepteridine^TM^ on the gelatinase activity of MMP-9 toward an insoluble collagen matrix. For these studies, we bacterially expressed recombinant human His-tagged proMMP-9cat comprising amino acid residues 20-447 (UniProtKB accession number: P14780) with intact pro-peptide domain (Ala20-Arg106), catalytic domain (Phe107-Gly223), fibronectin domain (Asn224-Cys388), and zinc-binding domain (Pro389-Pro477).

**Figure 4:**
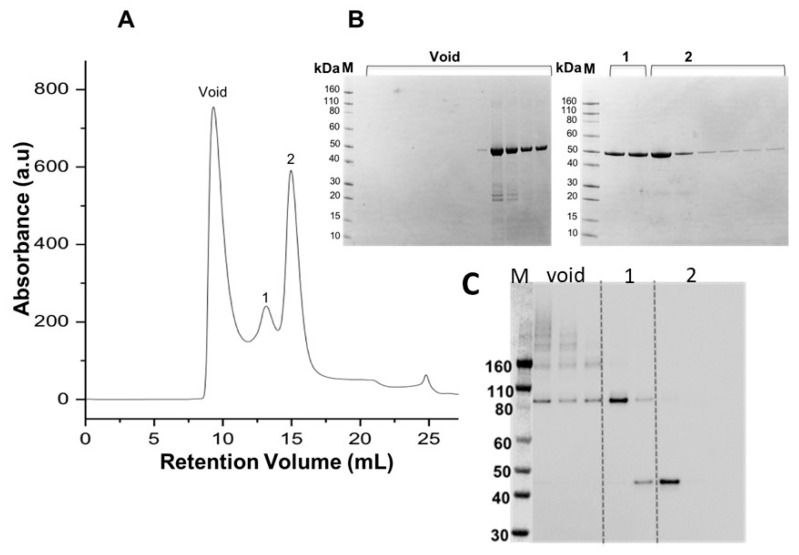
Purification of monomeric His-tagged proMMP-9cat. (**A**) Size exclusion chromatography performed in 20 mM Tris-HCl (pH 8.0), 150 mM NaCl, and 0.1 mM TCEP. (**B**) SDS-PAGE fraction analyses under reducing conditions. (**C**) Corresponding Western blot analyses from SDS/PAGE performed under non-reducing conditions with an anti-MMP-9 monoclonal antibody and chemiluminescent detection.

Figure 4 shows the elution profile of His-tagged proMMP-9cat following denaturing purification from inclusion bodies and IMAC chromatography coupled with on-column folding. All three peaks from size exclusion chromatography (Figure 4A) correspond to the expected molecular mass of the His-tagged proMMP-9cat monomer (M_W_~48 kDa), as assessed by Coomassie blue staining SDS-PAGE (Figure 4B). Western blot analyses from SDS/PAGE performed under non-reducing conditions using an anti-MMP-9 monoclonal antibody indicated the presence of higher M_W_ bands (>160 kDa and 80 kDa) for His-tagged proMMP-9cat eluted within the void peak and peak one, consistent with improperly folded or large aggregated structures. By comparison, peak two, from size exclusion chromatography, showed a band around 48 kDa, consistent with the expected molecular mass of the His-tagged proMMP-9cat monomer.

**Figure 5:**
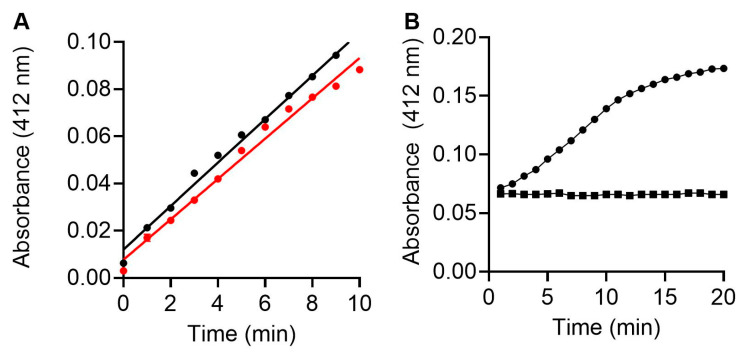
Activity of MMP-9cat. (**A**) Activity of MMP-9cat (black) compared to commercial MMP-9 (red). The assay was read at A412 nm. (**B**) Inhibition of MMP-9cat by NNGH. MMP-9cat was incubated with (■) or without (●) 6.5 µM NNGH for 1 h prior to initiation of the hydrolysis reaction via the addition of the thiopeptide chromogenic substrate. Each data point shows mean ± SEM (n = 3).

We next investigated the catalytic ability of MMP-9cat lacking the pro-domain to hydrolyse the thiopeptide chromogenic substrate (100 μM) (Figure 5). The specific activity of MMP-9cat (0.186 U/µg) was identical within experimental error to commercial MMP-9 (0.2 U/µg) (Figure 5A). Incubation of MMP-9cat with NNGH [35] for 1 h resulted in complete inhibition (Figure 5B), consistent with chelation of the active site zinc cation.

### 3.4. Gelatin Zymography Assessment of Lepteridine^TM^ and Its Ability to Inhibit His-Tagged proMMP-9cat and MMP-9cat Activity

We utilized gelatin zymography to assess the effect of Lepteridine^TM^ on the gelatinolytic activity of His-tagged proMMP-9cat and MMP-9cat. Unlike the fluorescent and colorimetric based assays that rely on small soluble substrate cleavage, the fibronectin type II domain is essential for gelatinolytic activity as it anchors MMP-9 to the gelatin matrix [43]. Gelatin zymography also relies on the initial denaturation of proMMP-9cat by SDS during electrophoresis, followed by its renaturation via progressive removal of SDS from the gel with Triton X-100 [44]. This refolding process exposes the proMMP-9cat active site and autoactivates a proportion of proMMP-9cat without cleaving the pro-domain [45]. Thus, measurable gelatinolytic activity of proMMP-9cat (48 kDa) is commonly observed in gelatin zymography [46,47,48].

**Figure 6:**
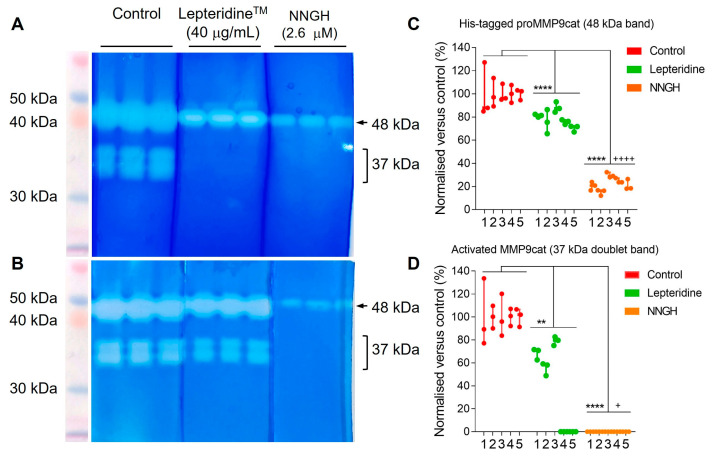
Lepteridine^TM^ inhibits His-tagged proMMP-9cat and MMP-9cat gelatinase activity. (**A**,**B**) Two representative gelatin zymography experiments showing His-tagged proMMP-9cat and MMP-9cat contained within the gels incubated in normal developing buffer (control) and buffer supplemented with Lepteridine^TM^ (40 µg/mL) and NNGH (2.6 µM). Each experiment comprised triplicate technical replicates for each of the control, Lepteridine^TM^, and NNGH treatments. Top clear band at 48kDa represents gelatinase activity from autoactivated His-tagged proMMP-9cat with pro-peptide domain attached. Bottom doublet bands at ~37 kDa represent gelatinase activity from MMP-9cat with fully cleaved pro-peptide domain. (**C**,**D**) Quantitative densitometric analyses of 48 kDa and 37 kDa doublet bands, respectively, from five separate zymography experiments (1–5). Data points represent Lepteridine^TM^ and NNGH band intensities normalised to mean intensity of the control band’s technical replicates for each respective experiment. Significance was determined by one-way ANOVA, followed by Tukey’s multiple comparison test when the *p* value summary was <0.05. **** *p* < 0.0001, ** *p* < 0.01 vs. control; ^++++^ *p* < 0.0001, ^+^ *p* < 0.05 vs. Lepteridine^TM^ treatment.

For these experiments, we generated active MMP-9cat by treating His-tagged proMMP-9cat with trypsin (Section 2.8). This treatment retained a proportion of active His-tagged proMMP-9cat with attached pro-peptide domain, as indicated by retention of the 48 kDa band (Figure 6A,B). By comparison, the 37 kDa doublet bands represent activity of MMP-9cat with cleaved pro-peptide domain. We then compared the effect of 40 μg/mL Lepteridine^TM^ and the catalytic active site inhibitor, NNGH, on gelatin digestion by His-tagged proMMP-9cat and MMP-9cat compared to the control. Densitometric analyses indicated that Lepteridine^TM^ attenuated hydrolysis of the gelatin substrate by His-tagged proMMP-9ca (48 kDa) (Figure 6C) and MMP-9cat (37 kDa) (Figure 6D) by 22% ± 8 (mean ± SD, n = 5) and 59% ± 35 (mean ± SD, n = 5), respectively. By comparison, NNGH exerted more pronounced inhibition of His-tagged proMMP-9cat (78% ± 6 (mean ± SD, n =5)) and completely abolished MMP-9cat activity.

While the percentage inhibition of His-tagged proMMP-9cat (48 kDa band) by Lepteridine^TM^ was consistent across the five experiments (Figure 6C), the percentage inhibition of the activated form, MMP-9cat (37 kDa doublet bands), was variable (Figure 6D). The same amount of MMP-9 protein was loaded in control and treatment lanes and control band intensities were detectable in all the assays performed. In two of the five experiments performed, Lepteridine^TM^ completely ablated the densitometric signal (see representative gel, Figure 6A). This apparent lack of signal is likely due to band intensities falling below the dynamic range of detection for this assay, thereby skewing the overall mean percentage inhibition. This outcome could arise from differences in protein gel loading as well as decreased cleavage efficiency of His-tagged proMMP-9cat with trypsin, resulting in a decreased generated proportion of MMP-9cat. 

### 3.5. Gastric Stability of Lepteridine^TM^

**Figure 7:**
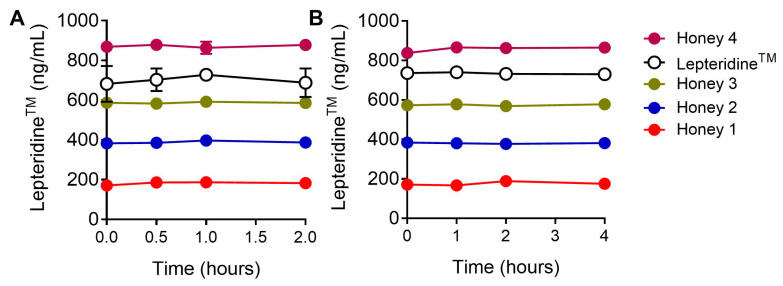
Simulated gastric (**A**) and intestinal (**B**) digestion does not affect the recovery of Mānuka honey-derived or -synthesised Lepteridine^TM^. Types 1–4 of Mānuka honey have different natural Lepteridine^TM^ concentrations. Each data point shows mean ± SEM (n = 3).

While Mānuka honey can be readily applied topically to treat wounds and other inflammatory skin conditions [4,6], in order to impact gastrointestinal inflammation, its active ingredients would need to be orally consumed and survive the process of digestion. Therefore, we investigated the stability of natural Lepteridine^TM^ in four types of Mānuka honey and chemically synthesized Lepteridine^TM^ in a simulated gastrointestinal digestion model. Mānuka honey with naturally occurring different concentrations of Lepteridine^TM^ or synthetic Lepteridine^TM^ was incubated under gastric and intestinal digestion conditions for 2 or 4 h, respectively. No significant degradation of Lepteridine^TM^, in either in its natural form in the four types of Mānuka honey or as a pure synthetic compound, was observed, with close to 100% recovery of Lepteridine^TM^ levels at all measured timepoints sampled during incubation (Figure 7).

## 4. Discussion

Mānuka honey is reported to have a range of bioactivities including anti-inflammatory properties. Lepteridine^TM^ is a natural pteridine derivative found uniquely in Mānuka honey; however, its biological activity is unknown. Via gelatin zymography assays, we showed the partial inhibition of MMP-9 gelatinase activity by Lepteridine^TM^ at concentrations comparable with those found naturally in Mānuka honey [17,49].

The molecular modelling analyses were equivocal with regard to interactions between Lepteridine^TM^ at the catalytic S1′ subsite or fibronectin domain. Nevertheless, the more pronounced partial inhibition of MMP-9 gelatinase activity by Lepteridine^TM^, as compared to the colorimetric (short peptide substrate) assay, provides some insight into the binding mechanisms involved. MMP-9, like MMP-2, possesses a different binding mechanism for short peptide substrates and gelatin. Short peptide substrates are designed to bind directly in or near the S1′ subsite cleft within the primary catalytic active site, which coordinates substrate binding and hydrolysis [50]. This mode of binding does not require the fibronectin type II domain [51,52]. The weak inhibitory effect of Lepteridine^TM^ (~10%) on MMP-9 activity towards the soluble chromogenic substrate is suggestive of binding interactions distinct from the catalytic active site where most zinc-chelating MMP inhibitors act [53]. It is not uncommon for exo site inhibitors to perturb binding affinity or bond cleavage of the substrate in a substrate-specific manner [54].

By comparison, the fibronectin II domain is essential for the binding and resulting digestive activity of MMP-9 towards gelatin [43,50]. The digestion of gelatin is a two-step process with separate binding and cleavage sites. The fibronectin II domain anchors MMP-9 to the gelatin matrix, which then facilitates further hydrolysis at the active site [51,52]. Interactions between Lepteridine^TM^ and the fibronectin II domain that perturb gelatin binding and subsequent hydrolysis of the gelatin substrate matrix are compatible with the observed inhibition of MMP-9 gelatinolytic activity.

While acknowledging the limitations of molecular modelling approaches [55], our analyses nevertheless indicate that Lepteridine^TM^ is not a ligand of MMP-9 in the classical sense. Rather, MMP-9 inhibition appears to be a consequence of the fragment-based nature of the Lepteridine^TM^ pteridine molecule, permitting low affinity and potentially non-specific binding interactions. The molecular dynamic simulations revealed a high dissociation rate from the fibronectin domain, indicating rapid exchange kinetic behaviour. This analyses therefore suggests that MMP-9 inhibition persists only while Lepteridine^TM^ concentrations are elevated sufficiently for binding interactions to occur. Speculatively, this mechanism of action might apply more broadly to other associated small molecule components present in Mānuka honey such as phenolic acids and flavonoids with fragment-like properties. The contribution of these small molecule components in the wound healing and broader anti-inflammatory actions of Mānuka honey is still largely unknown. One intriguing possibility is that they help mute inflammatory states through low affinity interactions with inflammatory mediators.

Mānuka honey reportedly decreases MMP-9 release from HL-60 (neutrophil) cells in vitro [56] and increases MMP-9 expression in diabetic foot ulcers, without affecting healing time [57]. However, the inhibitory effect of Mānuka honey or Lepteridine^TM^ on MMP-9 activity per se has not been reported previously. The observed inhibition of MMP-9 gelatinase activity by Lepteridine^TM^ may suggest scope for therapeutic intervention in vivo. This possibility could be further pursued by investigating the therapeutic utility of Lepteridine^TM^ in recalcitrant chronic wounds and more broadly in other gastrointestinal inflammatory disorders such as Crohn’s disease and gastric ulcer formation. In these pathologies, increased MMP-9 activity is associated with a pro-inflammatory state [58], which it can help sustain through proteolytic processing of inflammatory cytokines and chemokines into more active forms, such as pro-IL-1β and IL-8 [31,59].

The simulated digestion experiments indicate that Lepteridine^TM^ is stable under conditions in the stomach and gastrointestinal tract, suggesting it would not incur significant degradation during digestion. Consequently, oral consumption of Mānuka honey with endogenous Lepteridine^TM^ concentrations sufficient to inhibit MMP-9 is likely to retain bioavailability in vivo. Since Mānuka honey has a large range of natural variation in endogenous Lepteridine^TM^ concentrations (3–44 mg/kg) [17,33], testing Mānuka honey for Lepteridine^TM^ content would be necessary to establish standardised honey formulations to determine therapeutic efficacy and application.

The partial inhibition of MMP-9 gelatinase activity by Lepteridine^TM^ has potential clinical advantages over inhibitors that act via chelation with the active site zinc cation. For example, the MMP-9 inhibitor, NNGH, targets the primary catalytic domain and was highly effective in attenuating both gelatinolytic and peptidase activities of MMP-9cat. However, NNGH is not a viable therapeutic candidate as it is a broad-spectrum MMP inhibitor and can inhibit closely related enzymes including the “A disintegrin and metalloproteinase domain” (ADAM) family of proteases [60,61]. These enzymes have a diverse range of substrates including growth factors, cell adhesion molecules, angiogenic factors, chemokines, and cytokines. Consequently, uncontrolled inhibition can lead to various unwanted side effects [62,63].

While not a chelator, our modelling data suggest that Lepteridine^TM^ could potentially have a broad range of protein targets due to its fragment-based nature. Nevertheless, within the context of MMP-9 gelatinase activity, the milder inhibition observed with Lepteridine^TM^ as compared to NNGH, may be preferential for wound healing. Thus, while elevated MMP-9 activity is detrimental to the healing process, some MMP-9 activity is nevertheless still required to degrade irregular portions of the matrix to support healing. A mechanism whereby Lepteridine^TM^ exerts its actions through the fibronectin domain, which is restricted to MMP-2 and MMP-9, may also provide some desirable specificity.

Lepteridine^TM^ was discovered through the observation of an unexpected UV absorbance by HPLC when examining Mānuka honey for the presence of leptosperin [7]. Lepteridine^TM^ has a partially overlapping fluorescence spectra (ex/em: 330/470 nm) with the fluorescence product from the commercial assay (ex/em: 320/395nm). Since this overlap is small and peak emission spectra are well separated, the likelihood of intrinsic autofluorescence of Lepteridine^TM^ quenching the MMP-9 activity assay fluorescence product emission is unlikely. Regardless, the inability of Lepteridine^TM^ to attenuate the accumulation of MMP-9 cleaved peptide fragments, as detected by mass spectrometry, provides evidence that either Lepteridine^TM^ and/or its autofluorescence interferes with the fluorescence readout of the commercial substrate cleavage MMP-9 activity assay. Fluorescence interference is a common phenomenon in fluorometric analysis [64]. Since many natural compounds display autofluorescence and have complex matrices, this finding is a cautionary tale to undertake appropriate controls, and secondary assays to confirm results based on fluorescent activity, particularly in Mānuka honey research. 

## 5. Conclusions

This work provides evidence that the Mānuka honey unique pteridine derivative Lepteridine^TM^ likely survives the digestive tract when consumed orally. We identified MMP-9 as a putative target with demonstrated partial inhibition of gelatinase activity using gelatin zymography. Given the role of MMP-9 in a number of inflammatory diseases, including gastrointestinal inflammation, mucosal damage, and ulceration, this work has implications for understanding and further investigating the impact of Mānuka honey standardized for natural Lepteridine^TM^ content on gastrointestinal disorders.

## 6. Intellectual Property

- WO/2017/099612—Marker Compounds of Leptospermum Honeys and Methods of Isolation and Assaying Thereof.
- WO/2021/002763—Use of a Composition Comprising 3,6,7-Trimethyllumazine for Preventing, Ameliorating, or Treating MMP-9-Associated Conditions and Inflammation.
- Lepteridine^TM^ is a trademark of Comvita Limited for 3,6,7-trimethyllumazine. All references to the Lepteridine trademark in this article are with the authorisation of Comvita Limited.

## Figures and Tables

**Table 1:** Non-Covalent Binding Scores for Lepteridine_TM_ to Active Site of Candidate Inflammatory Mediators using Molecular Docking Simulation. Docking structures are Protein Data Bank (PDB) structure references. Compounds compared with Lepteridine^TM^ for similarity in candidate docking scores are listed in Figure A3. Abbreviations: Cyclooxygenase-2 (PTGS2), Matrix metalloproteinase (MMP), Aminopeptidase N (ANPEP), Heat shock protein 90 (H90), Janus kinase (JAK), Pyruvate carboxylase (PC), TGS2, Endothelin B receptor (EDNRB), transglutaminase 2 (TGM2).

Candidate	Docking Structure	GoldScore	ChemScore	Compound Similarity	Likely Binding, or Interacting with Inhibitor Residues/Domains
MMP9	6ESM	53.47	16.81	Active/Inactive	No
EDNRB	6K1Q	48.35	17.63	NA	Unclear
PTGS2	5IKR	44.33	11.77	Inactive	No
JAK1	6N7A	43.32	19.33	Active/Inactive	Weak
HSP90	5XRD	40.27	13.63	Inactive	Weak
PC	3BG3	37.94	11.39	Inactive	No
TGM2	1KV3	37.52	20.38	Active/Inactive	Weak
ANPEP	4FYT	36.05	15.03	Inactive	Weak

## Data Availability

The datasets generated for this study are available on request to the corresponding author.

## Appendix A

**Table A1:** Candidates for Lepteridine^TM^ docking modelling selection.

Candidate	Rationale	Citation
MMP-9	Key role in extracellular matrix remodelling, cell proliferation, and inflammatory pathways including cytokine and chemokine processing. Associated with gastric mucosal damage and ulcer formation.	[31,58,65]
EDNRB	Receptor for ET-1 produced by gastrointestinal tract. Evokes dysregulation of cytokine production and a feature of inflammatory bowel disease.	[66]
PTGS2	Intestinal immune response regulation.	[67]
JAK1	Receptor for several inflammatory cytokines. Target of some ulcerative colitis medications.	[68]
HSP90	Upregulated in inflammation and coordinates transcriptional response. Potential inflammatory bowel disease treatment target.	[69]
PC	Potential mediator of metabolic stress-driven inflammation and oxidative stress.	[70]
TGM2	Can regulate cytokine-induced gene transcription via nuclear factor-κB. Upregulated in an inflammatory state and wounds.	[71,72]
ANPEP	Removes amino acids from peptide substrates. Plays key roles in hormone regulation, maturation, and degradation of bioactive peptides including inflammatory cytokines.	[73]
